# Anaphylaxis following rivaroxaban ingestion: report of an extremely rare case

**DOI:** 10.11604/pamj.2021.38.333.28401

**Published:** 2021-04-06

**Authors:** Fahmi Yousef Khan, Theeb Osama Sulaiman

**Affiliations:** 1 Department of Medicine, Hamad General Hospital, Doha, Qatar

**Keywords:** Rivaroxaban, anaphylactic reaction, enoxaparin, deep vein thrombosis, a case report

## Abstract

We reported an anaphylactic reaction following ingestion of rivaroxaban in a 48-years-old male, who was recently discharged from the hospital as a case of deep vein thrombosis. At home, the patient developed a diffuse itchy skin rash, shortness of breath, and vomiting 30 minutes after rivaroxaban ingestion. Emergency Medical Service found that the patient had severe dyspnea, low blood pressure, and decreased blood oxygen saturation. The patient was given oxygen, intramuscular epinephrine, intravenous hydrocortisone, diphenhydramine, salbutamol nebulizer, and was immediately transferred to the emergency department of Hamad General Hospital. Subcutaneous enoxaparin was initiated, while hydrocortisone and salbutamol nebulizer continued. On the next day, his vital signs had stabilized, and intravenous hydrocortisone was switched to prednisolone tablets, and salbutamol nebulizer was switched to budesonide/salmeterol inhaler, whereas enoxaparin was overlapped with warfarin. After achieving the target international normalized ratio (INR), enoxaparin was discontinued and the patient was discharged with significant clinical and laboratory improvement.

## Introduction

In the treatment of patients with venous thromboembolism (VTE) and atrial fibrillation (AF), direct oral anticoagulants (DOACs) have overshadowed warfarin due to less intracranial bleeding, simplified dosing, and predictable pharmacokinetics compared to warfarin [1, 2]. Although, a growing number of reports of adverse drug reactions such as drug eruptions, urticaria, lichenoid eruption, and exanthematous rash have been published [2-4], anaphylaxis due to DOACs is extremely rare. Of 7111 participants in the rivaroxaban treatment arm of the ROCKET-AF study, anaphylaxis was found in 0.01% of cases [3]. In this article, we have reported anaphylaxis after taking rivaroxaban in a male patient, to increase clinician's awareness of this rare, but life-threatening complication of this drug.

## Patient and observation

A 48-years-old male with no previous chronic illness presented to ED with a 4-day history of left leg pain and swelling. The patient was diagnosed with deep vein thrombosis (DVT) by a lower limb ultrasound doppler. He received one dose of enoxaparin subcutaneously and was discharged to continue on rivaroxaban. The patient took the first dose of rivaroxaban at home, denied having taken any specific medication or food simultaneously. Within 30 minutes he developed a diffuse itchy skin rash, shortness of breath, and vomiting. The family called the Emergency Medical Service (EMS), which upon arrival found that the patient had severe dyspnea, low blood pressure (80/60mm/Hg), and decreased peripheral blood oxygen saturation (SpO2). They suspected anaphylaxis and started the patient on oxygen (through non-rebreathing mask), intramuscular epinephrine (1ml of 1: 10,000 solution), intravenous hydrocortisone (100mg), diphenhydramine (50mg), salbutamol nebulizer, and immediately transferred him to the emergency department (ED) of Hamad General Hospital.

Upon arrival to the ED, he was conscious, oriented, but dyspneic with generalized urticaria. The temperature was 36.8°C, pulse rate 95 beats/minute, respiratory rate 26/minute and his blood pressure (BP) was 90/60mm/Hg. His SpO_2_ was 95% on 5 liters O_2_ through a non-rebreathing mask. Chest examination showed diffuse bilateral rhonchi, and the rest of his examination was unremarkable. In the ED, he received intravenous fluid, intravenous hydrocortisone 100mg every 8 hours, and intravenous diphenhydramine 50mg every 8 hours. Also, he was started on salbutamol nebulizer, while the oxygen was continued through a nasal cannula. Initial investigations showed white blood cells of 11400/µL, hemoglobin 13.5gm/L, and platelets of 320000/µL. Serum creatinine was 131µmol/L; ALT 34 U/L, AST 41 U/L, and Ig E was 622 kunits/L (Normal: 0.00-114 kunits/L). The coagulation profile was within the normal limit.

The patient was admitted to the medical ward as a case of anaphylaxis secondary to rivaroxaban. The diagnosis was based on the immediate development of life-threatening acute allergy symptoms after rivaroxaban ingestion and the dramatic response to epinephrine and hydrocortisone given early. Enoxaparin was initiated in a therapeutic dose and hydrocortisone as well as salbutamol nebulizer continued. On the next day, his vital signs had stabilized, and intravenous hydrocortisone was switched to prednisolone tablets 40 mg daily and salbutamol nebulizer was switched to budesonide/salmeterol inhaler, whereas enoxaparin was overlapped with warfarin. On the 6^th^ day of admission, the INR was 2.4, and on the next day, it was 2.6. Enoxaparin was discontinued and the patient was discharged after significant clinical and laboratory improvement. The patient was scheduled for follow-up at the immunology and warfarin clinics.

## Discussion

Anaphylaxis is a life-threatening generalized or systemic allergic or hypersensitivity reaction that can be triggered by any agent that can activate mast cells or basophils. Foods, medications, insect stings, and injections of allergen immunotherapy are the most common identifiable causes of anaphylaxis [5, 6]. Since the introduction of DOACs to treat VTE and AF, several case reports have been published describing hypersensitivity to rivaroxaban, which is manifested in various forms of skin rashes [2-4]. Anaphylactic reaction following ingestion of rivaroxaban is extremely rare accounting for 0.01% of cases in one study [3]. Symptoms and signs of anaphylaxis usually appear within 5 to 30 minutes of exposure to a triggering stimulus, but sometimes it can take several hours to develop [5, 6]. The most common symptoms include generalized urticaria, angioedema, and respiratory symptoms. Other less common symptoms include dizziness, unconsciousness, and gastrointestinal symptoms [5-7]. Similarly, our patient developed the symptoms (itchy skin rash, shortness of breath, and vomiting) 30 minutes after rivaroxaban ingestion.

The proposed mechanism of drug-induced anaphylaxis involves the abrupt release of pro-inflammatory mediators with vasoactive properties such as histamine, tryptase, and prostaglandin-leukotriene mediators, which cause a systemic reaction that is often serious and affects many organs [5-7]. Depending on the underlying mechanisms, this reaction can be defined as immunological or non-immunological. An IgE-dependent or independent mechanism may mediate immune anaphylaxis, whereas non-immune anaphylaxis requires direct activation of mast cells [7, 8]. IgE-mediated anaphylaxis requires an initial exposure and sensitization by an allergen, followed by re-exposure to the antigen through ingestion, inhalation, parenteral administration or topically [8, 9]. Skin testing, in vitro IgE tests, or challenge test may be used to determine the stimulus causing the anaphylactic reaction. Non-IgE-mediated anaphylaxis, on the other hand, may occur on first exposure to an antigen because prior exposure and sensitization are not required [9]. Although skin test to rivaroxaban, was not available in our hospital, we considered rivaroxaban as the culprit based on the fact that the symptoms began 30 minutes after rivaroxaban ingestion, while the patient denied taking any medications or specific food simultaneously. In addition, elevated IgE level suggests that this anaphylactic reaction had IgE mediated mechanism with unclear sensitization pattern.

Anaphylaxis is primarily a clinical diagnosis. Clinical criteria for the diagnosis of anaphylaxis have been proposed by the Allergy and Infectious Diseases National Institute and the Anaphylaxis and food allergy Network, which considers anaphylaxis highly likely if one of the following three criteria is fulfilled: First, acute onset of the disease, which occurs within minutes to several hours and affects the skin, mucous tissue, or both, e.g. widespread itching, hives or redness, and swelling of the lips/tongue/uvula, in addition to at least one of the following: a) Respiratory symptoms such as dyspnea, wheeze/bronchospasm, stridor, reduced PEF, and hypoxemia; b) Reduced BP or associated symptoms of end-organ dysfunction such as collapse, syncope, and incontinence. Second, two or more of the following, which occurs rapidly within minutes to hours after the patient's exposure to a possible allergen: a) Skin-mucosal tissue involvement, such as widespread itching, hives or redness, and swelling of the lips/tongue/uvula; b) Respiratory symptoms such as dyspnea, wheeze/bronchospasm, stridor, reduced PEF, and hypoxemia; c) BP reduction or associated symptoms of end-organ dysfunction such as collapse, syncope, and incontinence; d) Continuing gastrointestinal symptoms such as crampy abdominal pain, and vomiting. Third, BP reduction after the patient's exposure to a known allergen within minutes to several hours. BP reduction is age-specific: a) In infants and children, BP reduction is defined as a decrease of more than 30% in systolic BP; b) In adults, it is defined as a systolic BP of less than 90 mmHg or a drop in a person's systolic BP greater than 30% from his baseline [10]. Based on these criteria, we consider anaphylaxis highly likely in our patient, and we considered rivaroxaban as the culprit drug, with a high probability of an IgE-mediated mechanism.

## Conclusion

Anaphylactic reaction following ingestion of rivaroxaban is extremely rare. Our case highlights that rivaroxaban can be a potential trigger for anaphylaxis, but it is probably underestimated because it is a new drug. Therefore, clinicians and other healthcare providers should consider this rare and serious adverse effect while explaining the management plan to the patient who is a candidate for rivaroxaban.
